# Associations among discrimination, genetic susceptibility to inflammation, and C-reactive protein

**DOI:** 10.1016/j.bbih.2026.101258

**Published:** 2026-05-14

**Authors:** Alisha A. Crump, Jemar R. Bather, Robert F. Krueger, Mariana Rodrigues, Emiko O. Kranz, Ester Villalonga-Olives, Adolfo G. Cuevas

**Affiliations:** a Department of Social and Behavioral Sciences, NYU School of Global Public Health, New York, NY, USA; b Department of Biostatistics, NYU School of Global Public Health, New York, NY, USA; c Department of Psychology, University of Minnesota, Minneapolis, MN, USA; d Department of Practice, Sciences and Health Outcomes Research, University of Maryland Baltimore, Baltimore, MD, USA

**Keywords:** Psychosocial stressors, Biological dysregulation, Biological stress, Psychoneuroimmunology, Social genomics, Internalizing, Epigenetics, Lifestyle genomics

## Abstract

Genetic liability to inflammation may lead to heightened perception of discrimination (gene-environment correlation) or interact with discrimination exposure to amplify inflammation risk (gene-environment interaction). The present study examined the interrelationship between genetic predisposition, self-reported discrimination exposure, and inflammatory phenotypes. Data came from 6344 participants in the 2016 Health and Retirement Study Venous Blood Study. Polygenic risk scores for high-sensitivity C-reactive protein (PRS-hsCRP) were derived from genome-wide association study summary statistics. Daily discrimination was assessed using the Everyday Discrimination Scale and hsCRP was quantified from serum and natural log-transformed for analysis. Linear regression models were fit using multiple imputation, and analyses were stratified by ethnicity. Perceived discrimination exposure was positively associated with ln(hsCRP) levels among European American (b = 0.06, 95% CI: 0.02 to 0.10, *p* = 0.005) and Hispanic participants (b = 0.11, 95% CI: 0.01 to 0.21, *p* = 0.033), even after adjusting for genetic liability. No significant association was observed among African American participants. Additionally, no association between PRS-hsCRP and discrimination was found in European American or Hispanic groups. Among African American participants, higher PRS-hsCRP was associated with lower reported discrimination (b = −0.07, 95% CI: −0.13 to −0.01, *p* = 0.025). No significant gene-environment interactions were found within each ethnic group. We found no evidence to support a gene-environment interaction or gene-environment correlation. Perceived discrimination may influence inflammation independently of genetic liability. Nonetheless, further research is needed to replicate and validate these findings.

## Highlights

• The study examined if discrimination is associated with C-reactive protein (CRP).
• Discrimination was linked to higher CRP in European American and Hispanic groups.
• Findings highlight the importance of identifying biobehavioral pathways.

## Abbreviations

hsCRP -: High-sensitivity C-reactive protein
VBS: Venous Blood Study
HRS -: Health and Retirement Study
rGE -: Evocative gene-environment correlation
SNPs -: Single-nucleotide polymorphisms
GWAS -: Genome-Wide Association Study

## 1 Introduction

Extensive research has documented the deleterious associations of discriminatory experiences with physical and mental health outcomes, with mounting evidence suggesting that chronic exposure to discrimination can trigger cascading biological processes that may contribute to disease development (Cuevas et al., 2020; Lewis et al., 2015; Williams et al., 2019). Among the biological pathways through which discrimination affect health, systemic inflammation has garnered particular attention as a key mechanistic link between psychosocial stress and adverse health outcomes (Cuevas et al., 2020; Lawrence et al., 2022; Lockwood et al., 2018; Simons et al., 2021). Inflammation is a central biological response to stress, involving the activation of immune cells and the release of cytokines, which can influence multiple organ systems over time. Chronic exposure to discrimination can provoke low-grade, subclinical inflammation, which, although often imperceptible in the short term, exerts noxious effects on multiple organ systems and contributes to long-term disease risk.

C-reactive protein (CRP), a widely used biomarker of chronic low-grade inflammation, is consistently found to be associated with perceived discrimination exposure (Lawrence et al., 2022; Cuevas et al., 2020). In fact, a systematic review and meta-analysis synthesizing 25 empirical studies on discrimination and four biomarker outcomes, cortisol, CRP, interleukin-6, and telomere length, found that perceived discrimination was consistently associated with CRP but not with the other markers. These associations are found across population groups. For example, in a study of Black women with lupus, those who reported greater exposure to racial discrimination showed higher levels of CRP (Martz et al., 2023) . Similarly, in a separate study among Puerto Rican adults, greater exposure to major lifetime perceived discrimination was associated with increased odds of elevated CRP levels (Cuevas et al., 2019). Beyond these individual-level associations, population-level disparities in inflammation are well documented. African American and Hispanic adults consistently show higher CRP levels compared to their European American counterparts, suggesting that discrimination may play a role in these differences (Albert et al., 2004; Lam et al., 2020). While these findings highlight the potential inflammatory effect of discrimination, much of the existing research has not accounted for the potential role of genetic liability. This is an important gap, as individual genetic predispositions may influence how people biologically respond to chronic social stressors like discrimination (Cuevas A. et al., 2021; 2023).

**Fig. 1:**
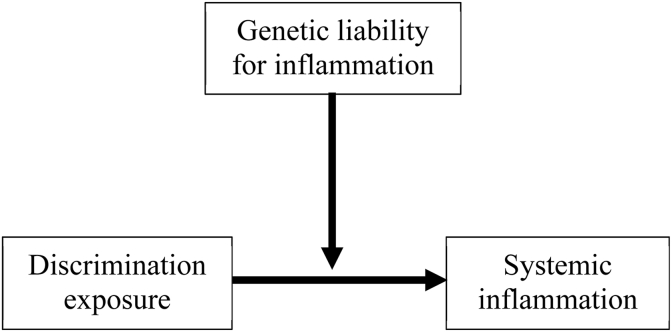
Diathesis-stress model framework of the potential moderating effect of genetic liability on the relationship between discrimination and systemic inflammation.

Two theoretical models offer complementary frameworks for understanding how genetic predisposition and environmental factors interact to shape health outcomes (Boardman et al., 2014; Burmeister et al., 2008; Ellis et al., 2011; Esposito et al., 2018; Reddon et al., 2016). The diathesis-stress model posits that individuals with a genetic predisposition to certain conditions may be especially susceptible to the harmful effects of environmental stressors, such as experiences of perceived discrimination (Belsky and Pluess, 2009; Sawyer et al., 2012). In the context of inflammation, those with genetic susceptibility to elevated inflammatory responses may exhibit heightened physiological reactivity when exposed to discrimination, thereby amplifying the risk of systemic inflammation (Fig. 1). More practically, this suggests that two individuals exposed to the same discriminatory event (e.g., a hostile workplace encounter, being denied housing, or receiving inferior medical care) might mount meaningfully different biological responses depending on their genetic background. For individuals with high inflammatory genetic liability, repeated discrimination could translate into chronically elevated inflammation faster and at lower cumulative exposure than it would for genetically lower-risk individuals, with downstream consequences for cardiometabolic disease, accelerated aging, and psychological well-being. Importantly, from an equity perspective, if genetic susceptibility amplifies the physiological cost of discrimination, then population groups who bear disproportionate burdens of discriminatory exposure may face a compounding biological disadvantage that environmental stress models alone cannot capture.

**Fig. 2:**
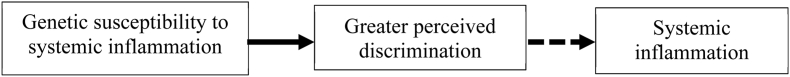
Evocative gene environment correlation model framework of the potential relationship between genetic susceptibility and discrimination exposure.

Complementing this model, the concept of evocative gene-environment correlation (rGE) offers an additional lens through which to examine the interplay between genetics and social experiences (Avinun, 2020; Avinun and Hariri, 2019; Böckerman et al., 2019; Jaffee and Price, 2007; Schnittker, 2010; South et al., 2015; Wilkinson et al., 2013). This model suggests that an individual's genetically influenced traits may elicit responses from their social environment, which in turn can shape their exposure to environmental stressors. Prior research has shown evidence of evocative rGE in the context of perceived discrimination and obesity, where individuals with a higher genetic risk for obesity were more likely to report discriminatory treatment, particularly among European Americans (Cuevas et al., 2023). Applied to inflammation, it is plausible that individuals with a high genetic liability for inflammation may have elevated basal inflammatory levels that influence how they interpret and respond to their social environment (Eisenberger et al., 2017) (Fig. 2). For example, heightened inflammation may sensitize individuals to perceive greater social threat, potentially increasing reports of discrimination Genetic predisposition to inflammation and perceived discrimination could manifest through multiple pathways. Individuals with higher genetic liability to inflammation may exhibit elevated baseline inflammatory activity, which can influence neural circuits involved in stress reactivity, threat perception, and emotional regulation (cite) This heightened stress responsiveness may lead these individuals to perceive social interactions as more threatening, thereby increasing reported experiences of discrimination. Additionally, a predisposition to inflammation may contribute to earlier onset of disease or more visible illness or disability, which can, in turn, elicit stigma or discriminatory treatment from others (Flavia et al., 2023). Therefore, it remains important to understand whether higher genetic inflammatory load causes individuals to experience more discrimination, to perceive the same level of discrimination more acutely, or both. Evolutionary frameworks offer one lens on this question, suggesting that a lower threshold for detecting interpersonal threat may have been adaptive under conditions of chronic social adversity, with the inflammatory system functioning as an anticipatory defense mechanism against physical harm signaled by social danger (Cole SW, 2019; McDade, 2012). In stable, low-adversity environments, however, this same sensitivity becomes maladaptive, producing chronic low-grade inflammation without the acute threat that would have justified it (Hertzman, 1999; Miller et al., 2011). This context does not diminish the real harm such dysregulation causes under chronic social stress; rather, it underscores why addressing upstream social determinants of discrimination remains essential alongside any consideration of biological susceptibility. Despite the relevance of these two well-established models, they have rarely been applied to understanding the relationship between perceived discrimination and inflammation. Integrating genetic susceptibility with social stress exposure holds promise for clarifying the mechanisms through which discrimination becomes biologically embedded.

The present study addresses a critical gap in the literature by examining the interrelationship between genetic susceptibility to inflammation, perceived discrimination, and inflammatory phenotype across European American, African American, and Hispanic populations. First, we assess whether genetic predisposition and perceived discrimination exposure interact to predict inflammation. Next, we test the gene-environment correlation hypothesis, which posits that individuals with greater genetic susceptibility may also report higher levels of perceived discrimination. By testing these models, this study offers a more inclusive investigation of the interplay between genetic liability, perceived discrimination, and inflammation, contributing to a deeper understanding of the biobehavioral pathways underlying existing population health profiles.

## 2 Materials and methods

### 2.1 Study design and setting

*Data come* from the Health and Retirement Study (HRS), one of the largest cohort studies of older adults in the United States (Sonnega et al., 2014). The HRS is a cooperative agreement between the National Institute on Aging and the University of Michigan (NIA U01AG009740) that began enrollment in 1992 with a nationally representative sample of noninstitutionalized adults born between 1931 and 1941 (ages 51-61 at baseline). The study employs a multistage, national area-clustered probability design with oversampling of Black individuals, Hispanic individuals (primarily Mexican Americans), and Florida residents to increase representation of racial/ethnic minorities. The HRS uses mixed-mode interviews, with participants alternating between in-person and telephone interviews biennially. New cohorts are added every six years to maintain representativeness as the original cohort ages. Details regarding the broader HRS methodology have been described elsewhere (Sonnega et al., 2014).

In 2016, the HRS launched the Venous Blood Study (VBS) to examine associations between psychosocial factors, environmental exposures, and biological markers (Nazmi and Victora, 2007). Participants were eligible for the VBS if they had completed their most recent HRS interview independently (without proxy assistance), were living in community settings rather than nursing facilities, and provided informed consent for blood collection. Among those meeting eligibility requirements, approximately 80% agreed to participate, with 83% of consenting participants successfully providing blood samples. Each participant received $50 for their contribution. This analysis received an exemption from the New York University Institutional Review Board given the use of de-identified, publicly accessible data. Our reporting follows the Strengthening the Reporting of Observational Studies in Epidemiology guidelines (von Elm et al., 2007).

### 2.2 Analytic sample

Of the 9934 VBS participants, we applied several exclusion criteria to create our analytic sample. We excluded 745 participants with zero sampling weights (indicating ineligibility for population-based inference), 1831 participants with three or more missing responses on discrimination items, and 1014 participants without available genetic ancestry information. These exclusions resulted in a final unweighted analytic sample of 6344 participants: European American (n = 4696; 74.0%), African American (n = 919; 14.5%), and Hispanic (n = 729; 11.5%).

### 2.3 Measurement of high-sensitivity C-reactive protein

Approximately two months following their 2016 HRS interview, VBS participants provided blood specimens through trained phlebotomists (Thyagarajan et al., 2018). Blood samples were processed via centrifugation and transported under refrigerated conditions to the University of Minnesota Advanced Research and Diagnostic Laboratory for analysis. High-sensitivity C-reactive protein (hsCRP) was quantified in serum using a latex-particle enhanced immunoturbidimetric assay (Roche Diagnostics, Indianapolis, IN) and analyzed on the Roche COBAS 6000 Chemistry platform. The clinical reference range for hsCRP was 0-5 mg/L, with laboratory inter-assay coefficients of variation of 5.1% at 1.05 mg/L and 6.7% at 3.12 mg/L. For analysis, hsCRP values were natural log-transformed to address positive skewness in the distribution.

HsCRP was selected as the primary inflammatory marker given its established associations with cardiovascular disease risk and its widespread use in population-based research (Ridker, 2003; Pearson et al., 2003). It is important to note, however, that CRP reflects the cumulative output of the inflammatory cascade and is not specific to any single inflammatory pathway or etiology (Ridker, 2016). While this limits mechanistic precision, hsCRP's stability, reliability, and clinical relevance across diverse populations make it a practical and validated marker for examining the impact of discrimination on systemic inflammation in large epidemiological samples (Ridker, 2003).

#### 2.3.1 Polygenic risk score development

Polygenic risk scores (PRSs) for hsCRP were derived from summary statistics obtained through the Genetic Investigation of Anthropometric Traits consortium, a large-scale genome-wide association study (Locke et al., 2015; Shungin et al., 2015). Each PRS was calculated as the sum of trait-associated single-nucleotide polymorphisms (SNPs) multiplied by their respective effect sizes from the discovery analysis:

$$\mathrm{PR}S_{k}=\sum \beta_{i}SNP_{ik}\text{,}$$

where i denotes individual SNPs linked to trait k. An inclusive p-value threshold of 1.0 was applied to incorporate all available SNPs meeting quality standards. Restricting the PRS to genome-wide significant SNPs alone risks excluding a substantial proportion of heritable signal, particularly for traits like CRP where common variants of small effect may explain meaningful variance (Ware et al., 2018). By retaining all SNPs, the PRS captures aggregate genetic liability and has greater predictive validity for continuous inflammation outcomes in population-based samples (Dudbridge, 2013; Wray et al., 2013).

Detailed protocols for HRS genetic data collection and processing have been published previously (Ware et al., 2018). In summary, participants were genotyped using Illumina Human Omni 2.5 BeadChip arrays (versions 2.5-4v1 and 2.5-8v1), capturing approximately 2.4 million genetic variants. Standard quality control procedures were implemented, removing individuals with chromosomal irregularities or familial relationships, and excluding variants with Hardy-Weinberg equilibrium deviations (*p* < 0.0001), uncertain allelic orientation, or excessive missingness (>5%). Genetic imputation was performed using the 1000 Genomes Project reference dataset (phase 3, version 5), expanding from 1,905,968 successfully genotyped variants to approximately 21 million imputed genetic markers.

### 2.4 Perceived daily discrimination

The Everyday Discrimination Scale assessed participants' frequency of discriminatory experiences (Williams et al., 1997). As part of the HRS Psychosocial and Lifestyle Questionnaire administered in 2010 or 2012 (depending on the participant's interview schedule), respondents reported how often they encountered unfair treatment in daily situations. The scale comprised six items addressing different forms of interpersonal discrimination: being treated with less courtesy or respect than others, receiving inferior service at restaurants or stores, others acting as though the respondent lacks intelligence, others behaving fearfully toward the respondent, experiencing threats or harassment, and receiving substandard medical care relative to others. Responses were recorded on a 6-point scale ranging from *1 (almost every day)* to *6 (never)*. Scores were reverse-coded and averaged to create a composite measure, with higher values indicating more frequent discrimination experiences (Cronbach's α = 0.82).

### 2.5 Covariates

Analyses adjusted for demographic characteristics and population structure. Demographic variables included age (continuous, in years), sex (male versus female), and body mass index (BMI; categorized as <18 or >30 kg/m^2^ versus 18-30 kg/m^2^). Five principal components (PC1 5 A through PC1 5 E) were included as continuous variables to control for population stratification within genetic analyses.

Age, sex, and BMI were included as established confounders of systemic inflammation (Ridker, 2003; Pearson et al., 2003). The five genetic PCs (PC1–PC5), derived from genome-wide SNP data, were included to control for systematic differences in allele frequencies across participants that reflect underlying ancestral heterogeneity rather than the genetic variants of interest. Failure to adjust for population stratification is a well-documented source of spurious associations in genome-wide and candidate gene studies, and inclusion of the top principal components is the standard analytic approach for addressing this confounding in genetic association analyses (Price et al., 2006).

### 2.6 Statistical analysis

Participant characteristics were summarized using means and SDs for continuous variables and frequencies and percentages for categorical variables. Between-group differences were assessed using Kruskal-Wallis tests for continuous variables and Pearson's chi-squared tests for categorical variables.

To test whether genetic predisposition to inflammation was associated with perceived daily discrimination exposure, we examined discrimination as the outcome with polygenic risk scores for hsCRP as the primary exposure. This tested the gene-environment correlation hypothesis that individuals with higher genetic susceptibility also experience more perceived daily discrimination. Next, we examined ln(hsCRP) as the outcome to test whether genetic and environmental factors interact to contribute to variation in inflammation. Models included main associations and systematic interaction terms to identify potential effect moderation between genetic predisposition and environmental exposures. Following best practices for gene-environment interaction analyses (Keller, 2014), we included interaction terms between polygenic risk scores, discrimination, and the covariates. This approach helps ensure any observed interactions between genetic predisposition and discrimination are not confounded by the covariates and reflect a more accurate estimate of the gene-environment interaction. All analyses were further stratified by ethnicity (European American, African American, and Hispanic). Sensitivity analyses excluding BMI were performed for all models.

Results from Little's missing completely at random (MCAR) test indicated that the data were not MCAR ($\chi_{\left(359\right)}^{2}$ = 928.42, *p* < 0.001; n = 6344 observations, 212 incomplete cases, 23 missing data patterns), leading us to reject the null hypothesis of MCAR. Under the assumption of missing at random (MAR), such that missingness is conditionally independent of unobserved values given observed data, we employed multivariate imputation by chained equations (MICE) using the *mice* R package (van Buuren and Groothuis-Oudshoorn, 2011). MICE was preferred over full information maximum likelihood given the presence of mixed variable types. Predictive mean matching was used for ln(hsCRP) and logistic regression for BMI. All analytic variables were included as predictors in the imputation models, with the exception of survey weights, which were excluded from imputation but applied in all regression analyses using VBS-specific weights to ensure generalizability. Estimates from regression analyses were pooled across ten imputed datasets using Rubin's rules (Rubin, 1987). All model assumptions (linearity, homoscedasticity, normality of residuals, and influential observations) were checked and met. Statistical significance was set at a two-sided *p* < 0.05. Analyses were conducted using R version 4.5.1 (R Core Team, R Foundation for Statistical Computing).

## 3 Results

### 3.1 Participant characteristics

The overall sample included 6344 adults (mean age 67.5 years, SD 9.4), 59.3% female, and 60.5% with BMI 18-30 kg/m^2^ (Table 1). European American participants were older on average (68.7 ± 9.4 years) compared to African American (64.4 ± 8.4 years) and Hispanic participants (64.0 ± 8.5 years; *p* < 0.001). African American participants had a higher proportion of females (67.1%) compared to European American (57.6%) and Hispanic participants (60.2%; *p* < 0.001). BMI distributions also varied significantly, with over half of African American participants (51.6%) having BMI outside the normal range (<18 or >30 kg/m^2^), compared to approximately one-third of European American participants (35.8%) and two-fifths of Hispanic participants (42.1%; *p* < 0.001).

**Table 1:** Study population characteristics, overall and stratified by ethnicity.

Characteristic	Overall	European American	African American	Hispanic	p-value^a^
	N = 6344	N = 4696	N = 919	N = 729	
**Age, Mean (SD)**	67.51 (9.37)	68.67 (9.40)	64.40 (8.44)	63.98 (8.52)	**<0.001**
**Sex, No. (%)**					**<0.001**
*Male*	2583 (40.7%)	1991 (42.4%)	302 (32.9%)	290 (39.8%)	
*Female*	3761 (59.3%)	2705 (57.6%)	617 (67.1%)	439 (60.2%)	
**Body mass index, No. (%)**					**<0.001**
*<18 or >30*	2463 (38.8%)	1682 (35.8%)	474 (51.6%)	307 (42.1%)	
*18 to 30*	3836 (60.5%)	3009 (64.1%)	444 (48.3%)	383 (52.5%)	
*Missing*	45 (0.7%)	5 (0.1%)	1 (0.1%)	39 (5.3%)	
**Polygenic risk score for high-sensitivity C-reactive protein, Mean (SD)**		−0.02 (0.99)	0.01 (0.97)	−0.01 (0.95)	0.40
**Daily discrimination, Mean (SD)**	1.54 (0.69)	1.51 (0.64)	1.70 (0.83)	1.55 (0.78)	**<0.001**
**High-sensitivity C-reactive protein (mg/L), Mean (SD)**	4.87 (11.66)	4.53 (11.53)	6.24 (11.84)	5.38 (12.14)	**<0.001**
*Missing*	51 (0.8%)	39 (0.8%)	10 (1.1%)	2 (0.3%)	

a Kruskal-Wallis rank sum test; Pearson's Chi-squared test.

Regarding the primary study variables, African American participants reported the highest levels of perceived daily discrimination (1.7 ± 0.8), followed by Hispanic (1.6 ± 0.8) and European American participants (1.5 ± 0.6; *p* < 0.001). Similarly, hsCRP levels were highest among African American participants (6.2 ± 11.8 mg/L), followed by Hispanic (5.4 ± 12.1 mg/L) and European American participants (4.5 ± 11.5 mg/L). In contrast, polygenic risk scores for hsCRP did not differ significantly across groups (*p* = 0.40).

### 3.2 Gene-environment correlation: polygenic risk and discrimination exposure

Table 2 shows the results from testing whether genetic predisposition to inflammation was associated with perceived daily discrimination exposure (gene-environment correlation). Among African American participants, higher polygenic risk scores for hsCRP were significantly associated with a 0.07 decrease (95% CI: −0.13, −0.01, *p* = 0.025) in perceived daily discrimination scores. No significant associations were observed among European American (b = 0.00, 95% CI: −0.03, 0.02, *p* = 0.76) or Hispanic participants (b = 0.03, 95% CI: −0.03, 0.10, *p* = 0.32). All models adjusted for age, sex, BMI, and genetic principal components.

**Table 2:** Adjusted associations between polygenic risk score for high-sensitivity C-reactive protein and daily discrimination among participants from the Health and Retirement Study, stratified by ethnicity.

*Characteristic*	European American (n = 4696)			African American (n = 919)			Hispanic (n = 729)		
	*b*	*95% CI*	*p value*	*b*	*95% CI*	*p value*	*b*	*95% CI*	*p value*
(Intercept)	2.53	(2.40, 2.66)	<0.001	2.58	(2.17, 2.99)	<0.001	2.23	(1.84, 2.63)	<0.001
Polygenic risk score hsCRP	0.00	(-0.03, 0.02)	0.76	−0.07	(-0.13, −0.01)	0.025	0.03	(-0.03, 0.10)	0.32
PC1 5 A	−1.52	(-3.76, 0.72)	0.18	−0.78	(-3.66, 2.10)	0.60	2.43	(-0.46, 5.33)	0.10
PC1 5 B	0.80	(-1.22, 2.82)	0.44	−2.38	(-5.47, 0.71)	0.13	0.34	(-2.70, 3.38)	0.83
PC1 5C	0.51	(-1.87, 2.89)	0.67	−1.22	(-4.09, 1.66)	0.41	3.53	(-1.39, 8.45)	0.16
PC1 5D	0.93	(-1.13, 2.99)	0.38	2.23	(-0.83, 5.29)	0.15	0.02	(-2.72, 2.76)	0.99
PC1 5 E	−0.29	(-2.54, 1.97)	0.80	4.23	(0.77, 7.69)	0.017	0.30	(-2.93, 3.53)	0.86
Age	−0.013	(-0.014, −0.011)	<0.001	−0.010	(-0.016, −0.004)	0.001	−0.010	(-0.016, −0.004)	0.002
Female sex (vs. Male)	−0.10	(-0.13, −0.06)	<0.001	−0.10	(-0.22, 0.01)	0.086	−0.03	(-0.14, 0.08)	0.59
BMI = 18 to 30 (vs. <18 or >30)	−0.16	(-0.20, −0.12)	<0.001	−0.25	(-0.36, −0.14)	<0.001	−0.05	(-0.18, 0.07)	0.41

Results were pooled across 10 multiple imputed datasets.

hsCRP = High-sensitivity C-reactive protein; PC = principal component; BMI = body mass index.

### 3.3 Gene-environment interactions and high-sensitivity C-reactive protein

#### 3.3.1 European American participants

**Fig. 3:**
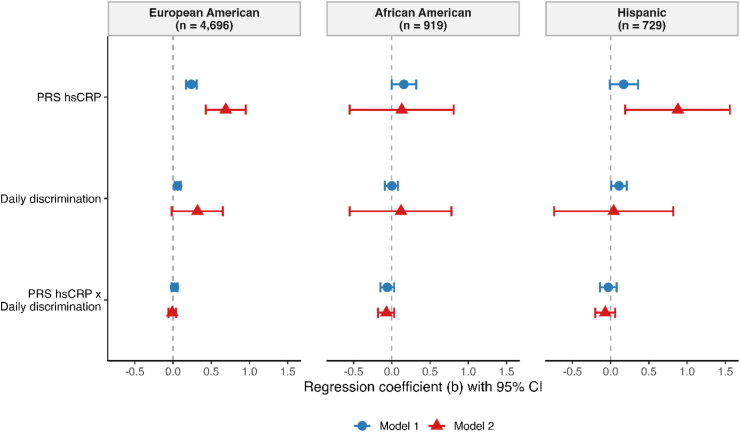
Forest plots showing the associations of polygenic risk score for high-sensitivity C-reactive (PRS hsCRP), daily discrimination, and the interaction between PRS hsCRP and daily discrimination with log-transformed high-sensitivity C-reactive protein levels.

Among European American participants (Table 3, Fig. 3), both genetic and environmental factors showed substantial associations with hsCRP levels. Higher polygenic risk scores for hsCRP were associated with markedly elevated inflammation in both the main associations model (b = 0.24, 95% CI: 0.17, 0.31, *p* < 0.001) and the interaction model (b = 0.69, 95% CI: 0.43, 0.95, *p* < 0.001). Greater exposure to perceived daily discrimination was also associated with increased hsCRP levels (b = 0.06, 95% CI: 0.02, 0.10, *p* = 0.005), though this association's significance was attenuated in the comprehensive interaction model (b = 0.32, 95% CI: −0.02, 0.65, *p* = 0.063). The hypothesized gene-environment interaction between polygenic risk and perceived daily discrimination showed no evidence of effect moderation in either model (main associations model: b = 0.02, 95% CI: −0.02, 0.06, *p* = 0.41; interaction model: b = −0.01, 95% CI: −0.06, 0.04, *p* = 0.81). Results from the sensitivity analysis excluding BMI did not substantially differ from those of the main analysis (Supplemental Table 1).

**Table 3:** Multiple linear regression analysis of log-transformed high-sensitivity C-reactive protein levels among 4696 European American participants: Unstandardized coefficients for demographic factors, polygenic risk scores, principal components, daily discrimination experiences, and interaction terms.

*Characteristic*	European American (n = 4696)					
	Model 1			Model 2		
	*b*	*95% CI*	*p value*	*b*	*95% CI*	*p value*
(Intercept)	0.62	(0.39, 0.84)	<0.001	0.24	(-0.29, 0.77)	0.38
Polygenic risk score hsCRP	0.24	(0.17, 0.31)	<0.001	0.69	(0.43, 0.95)	<0.001
Daily discrimination	0.06	(0.02, 0.10)	0.005	0.32	(-0.02, 0.65)	0.063
PC1 5 A	0.81	(-2.51, 4.14)	0.63	9.98	(-16.92, 36.89)	0.47
PC1 5 B	2.25	(-0.75, 5.24)	0.14	19.79	(-5.76, 45.35)	0.13
PC1 5C	15.83	(12.29, 19.36)	<0.001	36.71	(9.07, 64.35)	0.009
PC1 5D	−1.40	(-4.45, 1.66)	0.37	0.86	(-24.64, 26.36)	0.95
PC1 5 E	4.35	(1.02, 7.69)	0.010	31.62	(4.63, 58.62)	0.022
Age	0.005	(0.002, 0.008)	<0.001	0.011	(0.003, 0.019)	0.007
Female sex (vs. Male)	0.11	(0.06, 0.17)	<0.001	0.10	(-0.04, 0.24)	0.17
BMI = 18 to 30 (vs. <18 or >30)	−0.49	(-0.55, −0.43)	<0.001	−0.43	(-0.58, −0.28)	<0.001
Polygenic risk score hsCRP*Daily discrimination	0.02	(-0.02, 0.06)	0.41	−0.01	(-0.06, 0.04)	0.81
PC1 5 A*Age				0.07	(-0.28, 0.42)	0.69
PC1 5 A*Female				−0.59	(-7.35, 6.17)	0.86
PC1 5 A*BMI = 18 to 30				−2.33	(-9.71, 5.05)	0.54
PC1 5 A*Polygenic risk score hsCRP				0.32	(-3.26, 3.90)	0.86
PC1 5 A*Daily discrimination				−8.03	(-13.26, −2.79)	0.003
PC1 5 B*Age				−0.27	(-0.61, 0.07)	0.12
PC1 5 B*Female				5.58	(-0.48, 11.64)	0.071
PC1 5 B*BMI = 18 to 30				−1.50	(-7.94, 4.93)	0.65
PC1 5 B*Polygenic risk score hsCRP				−0.58	(-3.59, 2.44)	0.71
PC1 5 B*Daily discrimination				−1.05	(-5.94, 3.84)	0.68
PC1 5C*Age				−0.42	(-0.78, −0.05)	0.025
PC1 5C*Female				6.63	(-0.61, 13.88)	0.073
PC1 5C*BMI = 18 to 30				3.82	(-3.82, 11.46)	0.33
PC1 5C*Polygenic risk score hsCRP				1.96	(-1.17, 5.09)	0.22
PC1 5C*Daily discrimination				0.02	(-5.30, 5.35)	0.99
PC1 5D*Age				0.06	(-0.27, 0.40)	0.71
PC1 5D*Female				2.34	(-3.83, 8.52)	0.46
PC1 5D*BMI = 18 to 30				−1.61	(-8.36, 5.13)	0.64
PC1 5D*Polygenic risk score hsCRP				−1.05	(-4.22, 2.12)	0.52
PC1 5D*Daily discrimination				−4.61	(-9.46, 0.24)	0.063
PC1 5 E*Age				−0.43	(-0.78, −0.08)	0.015
PC1 5 E*Female				−3.72	(-10.53, 3.08)	0.28
PC1 5 E*BMI = 18 to 30				2.51	(-4.97, 10.00)	0.51
PC1 5 E*Polygenic risk score hsCRP				−0.57	(-4.19, 3.04)	0.76
PC1 5 E*Daily discrimination				1.59	(-3.71, 6.88)	0.56
Polygenic risk score hsCRP*Age				−0.006	(-0.010, −0.003)	<0.001
Polygenic risk score hsCRP*Female				0.04	(-0.03, 0.10)	0.30
Polygenic risk score hsCRP*BMI = 18 to 30				−0.03	(-0.10, 0.04)	0.33
Daily discrimination*Age				−0.004	(-0.009, 0.001)	0.15
Daily discrimination*Female				0.01	(-0.07, 0.09)	0.79
Daily discrimination*BMI = 18 to 30				−0.03	(-0.12, 0.05)	0.45

Results were pooled across 10 multiple imputed datasets.

hsCRP = High-sensitivity C-reactive protein; PC = principal component; BMI = body mass index.

#### 3.3.2 African American participants

Among African American participants (Table 4, Fig. 3), the pattern of associations differed markedly from European Americans. Polygenic risk scores for hsCRP showed a modest positive association with hsCRP levels in the main associations model (b = 0.158, 95% CI: −0.003, 0.319, *p* = 0.055) but showed little association in the interaction model (b = 0.13, 95% CI: −0.55, 0.81, *p* = 0.71). Perceived daily discrimination showed virtually no association with hsCRP levels in either model (main associations model: b = 0.00, 95% CI: −0.09, 0.08, *p* = 0.94; interaction model: b = 0.12, 95% CI: −0.55, 0.78, *p* = 0.73). As with European Americans, the gene-environment interaction between polygenic risk and perceived daily discrimination showed no meaningful effect moderation (main associations model: b = −0.06, 95% CI: −0.15, 0.03, *p* = 0.21; interaction model: b = −0.07, 95% CI: −0.18, 0.03, *p* = 0.18). Excluding BMI from the model did not meaningfully alter the results relative to the main analysis (Supplemental Table 2).

**Table 4:** Multiple linear regression analysis of log-transformed high-sensitivity C-reactive protein levels among 919 African American participants: Unstandardized coefficients for demographic factors, polygenic risk scores, principal components, daily discrimination experiences, and interaction terms.

*Characteristic*	African American (n = 919)					
	Model 1			Model 2		
	*b*	*95% CI*	*p value*	*b*	*95% CI*	*p value*
(Intercept)	1.71	(1.14, 2.29)	<0.001	1.38	(0.11, 2.64)	0.033
Polygenic risk score hsCRP	0.158	(-0.003, 0.319)	0.055	0.13	(-0.55, 0.81)	0.71
Daily discrimination	0.00	(-0.09, 0.08)	0.94	0.12	(-0.55, 0.78)	0.73
PC1 5 A	−0.97	(-4.70, 2.75)	0.61	−6.77	(-37.77, 24.23)	0.67
PC1 5 B	0.00	(-4.02, 4.02)	1.00	1.40	(-30.81, 33.62)	0.93
PC1 5C	2.73	(-1.04, 6.49)	0.16	−4.75	(-35.05, 25.56)	0.76
PC1 5D	3.23	(-0.78, 7.25)	0.11	11.81	(-19.28, 42.89)	0.46
PC1 5 E	−3.34	(-8.14, 1.45)	0.17	−14.74	(-50.11, 20.64)	0.41
Age	−0.006	(-0.014, 0.003)	0.18	0.00	(-0.02, 0.02)	0.88
Female sex (vs. Male)	0.10	(-0.05, 0.25)	0.21	−0.03	(-0.37, 0.31)	0.85
BMI = 18 to 30 (vs. <18 or >30)	−0.53	(-0.68, −0.38)	<0.001	−0.55	(-0.90, −0.20)	0.002
Polygenic risk score hsCRP*Daily discrimination	−0.06	(-0.15, 0.03)	0.21	−0.07	(-0.18, 0.03)	0.18
PC1 5 A*Age				0.07	(-0.36, 0.50)	0.74
PC1 5 A*Female				−2.22	(-10.76, 6.33)	0.61
PC1 5 A*BMI = 18 to 30				−9.37	(-17.56, −1.18)	0.025
PC1 5 A*Polygenic risk score hsCRP				−6.35	(-9.90, −2.81)	<0.001
PC1 5 A*Daily discrimination				2.97	(-1.59, 7.52)	0.20
PC1 5 B*Age				−0.12	(-0.58, 0.33)	0.59
PC1 5 B*Female				10.08	(1.35, 18.80)	0.024
PC1 5 B*BMI = 18 to 30				−1.42	(-10.06, 7.22)	0.75
PC1 5 B*Polygenic risk score hsCRP				−2.41	(-6.42, 1.59)	0.24
PC1 5 B*Daily discrimination				0.45	(-4.70, 5.61)	0.86
PC1 5C*Age				0.12	(-0.32, 0.56)	0.58
PC1 5C*Female				−4.65	(-12.69, 3.38)	0.26
PC1 5C*BMI = 18 to 30				−5.27	(-13.34, 2.79)	0.20
PC1 5C*Polygenic risk score hsCRP				−1.94	(-5.46, 1.57)	0.28
PC1 5C*Daily discrimination				3.31	(-1.75, 8.37)	0.20
PC1 5D*Age				−0.24	(-0.67, 0.19)	0.28
PC1 5D*Female				3.40	(-4.92, 11.73)	0.42
PC1 5D*BMI = 18 to 30				0.00	(-8.77, 8.77)	1.00
PC1 5D*Polygenic risk score hsCRP				−1.29	(-5.69, 3.12)	0.57
PC1 5D*Daily discrimination				2.94	(-2.43, 8.31)	0.28
PC1 5 E*Age				0.03	(-0.48, 0.54)	0.91
PC1 5 E*Female				4.34	(-6.02, 14.71)	0.41
PC1 5 E*BMI = 18 to 30				3.77	(-6.45, 13.98)	0.47
PC1 5 E*Polygenic risk score hsCRP				−2.38	(-6.21, 1.45)	0.22
PC1 5 E*Daily discrimination				1.80	(-3.82, 7.43)	0.53
Polygenic risk score hsCRP*Age				0.00	(-0.01, 0.01)	0.90
Polygenic risk score hsCRP*Female				0.07	(-0.10, 0.24)	0.42
Polygenic risk score hsCRP*BMI = 18 to 30				−0.05	(-0.22, 0.12)	0.59
Daily discrimination*Age				0.00	(-0.01, 0.01)	0.61
Daily discrimination*Female				0.07	(-0.10, 0.25)	0.42
Daily discrimination*BMI = 18 to 30				0.01	(-0.17, 0.20)	0.88

Results were pooled across 10 multiple imputed datasets.

hsCRP = High-sensitivity C-reactive protein; PC = principal component; BMI = body mass index.

#### 3.3.3 Hispanic participants

Among Hispanic participants (Table 5, Fig. 3), polygenic risk scores for hsCRP showed a modest positive association in the main associations model (b = 0.17, 95% CI: −0.01, 0.36, *p* = 0.065) and a stronger positive association in the interaction model (b = 0.88, 95% CI: 0.19, 1.56, *p* = 0.012). Greater perceived daily discrimination was associated with increased hsCRP levels in the main associations model (b = 0.11, 95% CI: 0.01, 0.21, *p* = 0.033) but showed little association in the interaction model (b = 0.04, 95% CI: −0.74, 0.82, *p* = 0.92). The gene-environment interaction showed no evidence of effect moderation (main associations model: b = −0.03, 95% CI: −0.14, 0.08, *p* = 0.60; interaction model: b = −0.07, 95% CI: −0.20, 0.06, *p* = 0.32). The main analysis findings were robust to the exclusion of BMI in sensitivity analyses (Supplemental Table 3).

**Table 5:** Multiple linear regression analysis of log-transformed high-sensitivity C-reactive protein levels among 729 Hispanic participants: Unstandardized coefficients for demographic factors, polygenic risk scores, principal components, daily discrimination experiences, and interaction terms.

*Characteristic*	Hispanic (n = 729)					
	Model 1			Model 2		
	*b*	*95% CI*	*p value*	*b*	*95% CI*	*p value*
(Intercept)	0.23	(-0.35, 0.81)	0.44	0.51	(-0.82, 1.85)	0.45
Polygenic risk score hsCRP	0.17	(-0.01, 0.36)	0.065	0.88	(0.19, 1.56)	0.012
Daily discrimination	0.11	(0.01, 0.21)	0.033	0.04	(-0.74, 0.82)	0.92
PC1 5 A	−5.25	(-9.14, −1.37)	0.008	7.58	(-26.59, 41.75)	0.66
PC1 5 B	2.12	(-1.95, 6.19)	0.31	23.97	(-8.43, 56.37)	0.15
PC1 5C	1.29	(-5.33, 7.90)	0.70	3.51	(-57.96, 64.98)	0.91
PC1 5D	−1.20	(-4.87, 2.47)	0.52	−5.07	(-36.27, 26.13)	0.75
PC1 5 E	2.64	(-1.68, 6.96)	0.23	4.38	(-30.10, 38.85)	0.80
Age	0.009	(0.001, 0.017)	0.029	0.00	(-0.02, 0.02)	0.98
Female sex (vs. Male)	0.20	(0.05, 0.35)	0.009	0.39	(0.04, 0.74)	0.028
BMI = 18 to 30 (vs. <18 or >30)	−0.31	(-0.47, −0.16)	<0.001	0.21	(-0.17, 0.58)	0.29
Polygenic risk score hsCRP*Daily discrimination	−0.03	(-0.14, 0.08)	0.60	−0.07	(-0.20, 0.06)	0.32
PC1 5 A*Age				−0.08	(-0.57, 0.42)	0.76
PC1 5 A*Female				−4.74	(-12.97, 3.49)	0.26
PC1 5 A*BMI = 18 to 30				−2.72	(-10.77, 5.34)	0.51
PC1 5 A*Polygenic risk score hsCRP				−0.58	(-5.21, 4.05)	0.81
PC1 5 A*Daily discrimination				−2.17	(-7.94, 3.60)	0.46
PC1 5 B*Age				−0.39	(-0.84, 0.05)	0.081
PC1 5 B*Female				5.93	(-2.38, 14.23)	0.16
PC1 5 B*BMI = 18 to 30				5.33	(-3.44, 14.11)	0.23
PC1 5 B*Polygenic risk score hsCRP				2.05	(-2.76, 6.87)	0.40
PC1 5 B*Daily discrimination				−4.39	(-10.42, 1.63)	0.15
PC1 5C*Age				−0.22	(-1.06, 0.63)	0.61
PC1 5C*Female				−4.05	(-18.15, 10.06)	0.57
PC1 5C*BMI = 18 to 30				6.63	(-7.21, 20.47)	0.35
PC1 5C*Polygenic risk score hsCRP				4.99	(-3.08, 13.05)	0.23
PC1 5C*Daily discrimination				8.58	(-2.09, 19.24)	0.12
PC1 5D*Age				0.03	(-0.42, 0.48)	0.89
PC1 5D*Female				−5.68	(-13.11, 1.74)	0.13
PC1 5D*BMI = 18 to 30				5.85	(-1.96, 13.67)	0.14
PC1 5D*Polygenic risk score hsCRP				−3.28	(-7.92, 1.37)	0.17
PC1 5D*Daily discrimination				1.60	(-3.36, 6.55)	0.53
PC1 5 E*Age				0.06	(-0.39, 0.52)	0.78
PC1 5 E*Female				−4.34	(-13.35, 4.67)	0.35
PC1 5 E*BMI = 18 to 30				−14.82	(-24.11, −5.53)	0.002
PC1 5 E*Polygenic risk score hsCRP				2.86	(-0.93, 6.65)	0.14
PC1 5 E*Daily discrimination				−0.65	(-7.87, 6.58)	0.86
Polygenic risk score hsCRP*Age				−0.01	(-0.02, 0.00)	0.21
Polygenic risk score hsCRP*Female				−0.28	(-0.47, −0.10)	0.003
Polygenic risk score hsCRP*BMI = 18 to 30				−0.18	(-0.37, 0.01)	0.070
Daily discrimination*Age				0.00	(-0.01, 0.02)	0.50
Daily discrimination*Female				−0.12	(-0.32, 0.08)	0.25
Daily discrimination*BMI = 18 to 30				−0.33	(-0.55, −0.11)	0.003

Results were pooled across 10 multiple imputed datasets.

hsCRP = High-sensitivity C-reactive protein; PC = principal component; BMI = body mass index.

## 4 Discussion

This study examined whether genetic predisposition to CRP plays a role in the association between perceived discrimination and CRP. We found no evidence that the association between perceived discrimination and CRP varied by polygenetic risk scores for PRS-CRP. We also found no evidence that PRS-CRP was associated with discrimination exposure.

We found no support for the gene-environment interaction hypothesis; that is, genetic susceptibility to inflammation did not moderate the association between perceived discrimination and CRP levels, suggesting that the physiological toll of discrimination may operate independently of one's genetic predisposition to inflammation. We found this to be the case in our study, as discrimination remained significantly associated with CRP for European and Hispanic individuals. The absence of an association among African American individuals may be partially explained by the pervasiveness of discrimination within this population. When exposure to discrimination is near-universal, it may account for between-group differences in CRP rather than driving variance within the group (Williams et al., 2003). Extending this reasoning, chronically elevated discrimination exposure may have shifted the inflammatory set point upward among African American participants such that additional variation in perceived discrimination produces diminishing marginal effects at the individual level. Nevertheless, genetic risk should not be overlooked. Among White participants, higher PRS-CRP was independently associated with elevated CRP, highlighting that genetic liability continues to be associated with inflammatory responses. The lack of association among Black and Hispanic participants is not surprising as it is likely due to the limitations of PRS derived from European populations (Logsdon et al., 2025; Petrovski and Goldstein, 2016). This issue with PRS, namely reduced predictive accuracy in non-European populations, remains a persistent challenge in genetics research (Duncan et al., 2019; Wang et al., 2022). This reduction in predictive validity is driven by several factors, including differences in linkage disequilibrium patterns, allele frequencies, and the underlying genetic architecture of complex traits across ancestral populations (Martin et al., 2019; Wojcik et al., 2019). As a result, the near-zero associations between PRS and CRP observed in Black and Hispanic participants in the current study likely reflect the limited transferability of the PRS rather than the absence of genetic contributions to inflammation in these groups. This limitation has important implications. Therefore, interpretation of the present findings for non-European populations should not be done without caveat. Future research should prioritize developing ancestry-specific PRS to more accurately assess genetic contributions to inflammation across diverse groups.

Several limitations should be considered when interpreting these findings. First, the cross-sectional design precludes causal inferences about the relationships between genetic factors, perceived discrimination, and inflammatory responses. An important caveat is that discrimination was assessed in 2010 or 2012 while hsCRP was measured in 2016, providing a four-to-six-year prospective window that lends modest support to the directionality of observed associations, making it more plausible that discrimination precedes elevated inflammation than the reverse. Nevertheless, we cannot rule out that earlier inflammatory status shaped subsequent discrimination perceptions, or that unmeasured confounders did not operate across this interval. Longitudinal studies with repeated measures of both constructs are needed to establish temporal relationships and examine how these associations may evolve over the life course. Second, the majority of Genome-Wide Association Studies (GWAS) used to inform the SNP weights came from GWAS on European ancestry groups and, as a result, PRSs for other groups may not have the same predictive capacity, transferability and interpretability (Ware et al., 2018). Third, beyond issues of cross-ancestry transferability, PRS carry additional limitations that warrant acknowledgment. PRS are derived from statistical associations between genetic variants and phenotypic outcomes, and as such, they are susceptible to environmental confounding (Mostafavi et al., 2020; Selzam et al., 2019). Furthermore, PRS may demonstrate poor generalizability across sociocultural and environmental contexts, even within broadly defined ancestral groups, limiting their interpretability in studies examining populations with diverse lived experiences (Ding et al., 2023). These constraints underscore the need for caution when using PRS as covariates or moderators in studies of social determinants of health, where genetic and environmental exposures are deeply intertwined. Future research should prioritize the development of ancestry-specific and diversity-informed PRS to more accurately and equitably assess genetic contributions to inflammation and other health outcomes across racially and ethnically diverse populations (Fatumo et al., 2022). Fourth, treating “Hispanic” as a single racial or genetic category is an important analytic limitation. The term encompasses individuals from diverse national-origin groups who vary substantially in Indigenous, European, and African ancestry, as well as in sociocultural contexts and in how they perceive and respond to discrimination. Because polygenic risk scores are primarily based on GWAS of European-ancestry populations, their application to admixed populations, including the Hispanic participants in this study, should be interpreted with caution. Future research should use ancestry-specific GWAS and, when sample sizes allow, disaggregate Hispanic subgroups to better capture within-group variation in both genetic risk and exposure to discrimination (Rao et al., 2025). Finally, the Everyday Discrimination Scale, while widely used and validated, may not capture all forms of discrimination relevant to health outcomes, particularly those that may be more salient for specific racial/ethnic groups. Future studies should consider incorporating multiple measures, including structural forms of discrimination, that may have different relationships with biological markers. Despite these limitations, this study represents an important contribution to the field by demonstrating the critical importance of stratified analyses in gene-environment research and providing novel insights into the complex relationships between genetic predisposition, perceived discrimination experiences, and inflammatory responses across diverse populations.

## 5 Conclusion

The current study highlights the relationships between genetic susceptibility, inflammation, perceived discrimination exposure, and inflammatory responses. We found no evidence for gene-environment interaction or gene-environment correlation, suggesting that discrimination remains an important social determinant of health that may operate independently of genetic liability. These findings challenge assumptions about the universality of gene-environment relationships in the context of discrimination and inflammation and underscore the need to better understand how discrimination as a social determinant “gets under the skin.”

## Clinical trial number

Not applicable.

## Funding

This work was supported by the 10.13039/100026007National Institute of 10.13039/100018696Health (10.13039/100000062National Institute of Diabetes and Digestive and Kidney Diseases): R01DK137246; R01DK137805. RFK was supported partly by 10.13039/100000002National Institutes of Health grants R01AG053217, R01AG077742, and U19AG51426.

## CRediT authorship contribution statement

**Alisha A. Crump:** Conceptualization, Writing – original draft, Writing – review & editing. **Jemar R. Bather:** Data curation, Formal analysis, Methodology, Writing – original draft, Writing – review & editing. **Robert F. Krueger:** Conceptualization, Writing – review & editing. **Mariana Rodrigues:** Writing – review & editing. **Emiko O. Kranz:** Writing – review & editing. **Ester Villalonga-Olives:** Writing – review & editing. **Adolfo G. Cuevas:** Conceptualization, Funding acquisition, Investigation, Methodology, Project administration, Supervision, Writing – original draft, Writing – review & editing.

## Declaration of competing interest

The authors declare the following financial interests/personal relationships which may be considered as potential competing interests:Adolfo Cuevas reports financial support was provided by National Institute of Diabetes and Digestive and Kidney Diseases. Robert Krueger reports financial support was provided by National Institute on Aging. If there are other authors, they declare that they have no known competing financial interests or personal relationships that could have appeared to influence the work reported in this paper.

## Data Availability

The data that support this study's findings are publicly available on the Health and Retirement Study website (https://hrs.isr.umich.edu/about).
