# Supplementary material for: Associations among discrimination, genetic susceptibility to inflammation, and C-reactive protein
Source: Brain Behav Immun Health. 2026 May 14;54:101258. doi: 10.1016/j.bbih.2026.101258 (PMC13213285; doi:10.1016/j.bbih.2026.101258)
Supplement: Multimedia component 1 [file mmc1.docx]

**Supplemental Table 1.** Multiple linear regression analysis of log-transformed high-sensitivity C-reactive protein levels among 4,696 European American participants: Unstandardized coefficients for demographic factors, polygenic risk scores, principal components, daily discrimination experiences, and interaction terms. These results are from sensitivity analyses excluding body mass index.

| *Characteristic* | *b* | *95% CI* | *p value* | *b* | *95% CI* | *p value* |  |
| --- | --- | --- | --- | --- | --- | --- | --- |
| (Intercept) | 0.34 | (0.11, 0.56) | 0.004 | -0.01 | (-0.55, 0.53) | 0.98 |  |
| Polygenic risk score hsCRP | 0.26 | (0.19, 0.33) | <0.001 | 0.72 | (0.46, 0.98) | <0.001 |  |
| Daily discrimination | 0.10 | (0.06, 0.15) | <0.001 | 0.34 | (-0.01, 0.68) | 0.053 |  |
| PC1 5A | 0.34 | (-3.07, 3.76) | 0.84 | 5.13 | (-21.95, 32.21) | 0.71 |  |
| PC1 5B | 1.88 | (-1.20, 4.96) | 0.23 | 21.31 | (-4.62, 47.23) | 0.11 |  |
| PC1 5C | 17.33 | (13.70, 20.97) | <0.001 | 42.82 | (14.69, 70.94) | 0.003 |  |
| PC1 5D | -0.92 | (-4.07, 2.23) | 0.57 | 2.54 | (-23.20, 28.28) | 0.85 |  |
| PC1 5E | 4.69 | (1.26, 8.12) | 0.007 | 34.80 | (7.70, 61.89) | 0.012 |  |
| Age | 0.00 | (0.00, 0.01) | 0.020 | 0.01 | (0.00, 0.02) | 0.027 |  |
| Female sex (vs. Male) | 0.14 | (0.08, 0.20) | <0.001 | 0.13 | (-0.01, 0.28) | 0.069 |  |
| Polygenic risk score hsCRP*Daily discrimination | 0.02 | (-0.02, 0.06) | 0.31 | 0.00 | (-0.05, 0.05) | 0.93 |  |
| PC1 5A*Age |  |  |  | 0.12 | (-0.24, 0.47) | 0.52 |  |
| PC1 5A*Female |  |  |  | -1.40 | (-8.32, 5.51) | 0.69 |  |
| PC1 5A*Polygenic risk score hsCRP |  |  |  | 0.76 | (-2.93, 4.46) | 0.69 |  |
| PC1 5A*Daily discrimination |  |  |  | -8.24 | (-13.56, -2.92) | 0.002 |  |
| PC1 5B*Age |  |  |  | -0.34 | (-0.69, 0.01) | 0.054 |  |
| PC1 5B*Female |  |  |  | 6.88 | (0.66, 13.10) | 0.030 |  |
| PC1 5B*Polygenic risk score hsCRP |  |  |  | -0.93 | (-3.99, 2.12) | 0.55 |  |
| PC1 5B*Daily discrimination |  |  |  | -0.37 | (-5.38, 4.65) | 0.89 |  |
| PC1 5C*Age |  |  |  | -0.45 | (-0.82, -0.08) | 0.018 |  |
| PC1 5C*Female |  |  |  | 7.62 | (0.22, 15.03) | 0.043 |  |
| PC1 5C*Polygenic risk score hsCRP |  |  |  | 1.84 | (-1.34, 5.03) | 0.26 |  |
| PC1 5C*Daily discrimination |  |  |  | -0.28 | (-5.72, 5.16) | 0.92 |  |
| PC1 5D*Age |  |  |  | 0.03 | (-0.31, 0.37) | 0.87 |  |
| PC1 5D*Female |  |  |  | 3.28 | (-3.05, 9.61) | 0.31 |  |
| PC1 5D*Polygenic risk score hsCRP |  |  |  | 0.04 | (-3.19, 3.26) | 0.98 |  |
| PC1 5D*Daily discrimination |  |  |  | -4.90 | (-9.82, 0.02) | 0.051 |  |
| PC1 5E*Age |  |  |  | -0.46 | (-0.82, -0.10) | 0.012 |  |
| PC1 5E*Female |  |  |  | -3.27 | (-10.25, 3.71) | 0.36 |  |
| PC1 5E*Polygenic risk score hsCRP |  |  |  | 0.39 | (-3.33, 4.12) | 0.84 |  |
| PC1 5E*Daily discrimination |  |  |  | 2.03 | (-3.35, 7.41) | 0.46 |  |
| Polygenic risk score hsCRP*Age |  |  |  | -0.01 | (-0.01, -0.01) | <0.001 |  |
| Polygenic risk score hsCRP*Female |  |  |  | 0.03 | (-0.03, 0.10) | 0.33 |  |
| Daily discrimination*Age |  |  |  | 0.00 | (-0.01, 0.00) | 0.16 |  |
| Daily discrimination*Female |  |  |  | 0.00 | (-0.08, 0.09) | 0.95 |  |
| Results were pooled across 10 multiple imputed datasets | | | | | | | |
| hsCRP = High-sensitivity C-reactive protein; PC = principal component | | | | | | | |

**Supplemental Table 2.** Multiple linear regression analysis of log-transformed high-sensitivity C-reactive protein levels among 919 African American participants: Unstandardized coefficients for demographic factors, polygenic risk scores, principal components, daily discrimination experiences, and interaction terms. These results are from sensitivity analyses excluding body mass index.

| *Characteristic* | *b* | *95% CI* | *p value* | *b* | *95% CI* | *p value* |
| --- | --- | --- | --- | --- | --- | --- |
| (Intercept) | 1.62 | (1.03, 2.21) | <0.001 | 1.17 | (-0.13, 2.47) | 0.077 |
| Polygenic risk score hsCRP | 0.19 | (0.03, 0.36) | 0.023 | 0.00 | (-0.70, 0.69) | 0.99 |
| Daily discrimination | 0.04 | (-0.05, 0.13) | 0.36 | 0.20 | (-0.48, 0.88) | 0.56 |
| PC1 5A | -1.03 | (-4.93, 2.86) | 0.60 | -13.03 | (-44.69, 18.63) | 0.42 |
| PC1 5B | -0.17 | (-4.30, 3.96) | 0.94 | -0.55 | (-33.50, 32.40) | 0.97 |
| PC1 5C | 3.42 | (-0.46, 7.30) | 0.084 | -4.27 | (-35.22, 26.68) | 0.79 |
| PC1 5D | 2.42 | (-1.92, 6.75) | 0.27 | 13.11 | (-19.29, 45.51) | 0.43 |
| PC1 5E | -3.71 | (-9.21, 1.79) | 0.18 | -11.24 | (-47.91, 25.43) | 0.55 |
| Age | -0.01 | (-0.02, -0.01) | 0.012 | 0.00 | (-0.02, 0.02) | 0.90 |
| Female sex (vs. Male) | 0.20 | (0.04, 0.35) | 0.013 | 0.04 | (-0.31, 0.38) | 0.83 |
| Polygenic risk score hsCRP*Daily discrimination | -0.06 | (-0.15, 0.03) | 0.21 | -0.07 | (-0.18, 0.04) | 0.20 |
| PC1 5A*Age |  |  |  | 0.02 | (-0.42, 0.45) | 0.94 |
| PC1 5A*Female |  |  |  | 1.12 | (-7.50, 9.73) | 0.80 |
| PC1 5A*Polygenic risk score hsCRP |  |  |  | -6.51 | (-10.11, -2.91) | <0.001 |
| PC1 5A*Daily discrimination |  |  |  | 4.41 | (-0.21, 9.02) | 0.061 |
| PC1 5B*Age |  |  |  | -0.08 | (-0.53, 0.38) | 0.74 |
| PC1 5B*Female |  |  |  | 8.71 | (0.04, 17.38) | 0.049 |
| PC1 5B*Polygenic risk score hsCRP |  |  |  | -1.50 | (-5.56, 2.55) | 0.47 |
| PC1 5B*Daily discrimination |  |  |  | -0.16 | (-5.34, 5.03) | 0.95 |
| PC1 5C*Age |  |  |  | 0.06 | (-0.38, 0.51) | 0.78 |
| PC1 5C*Female |  |  |  | -3.01 | (-11.25, 5.23) | 0.47 |
| PC1 5C*Polygenic risk score hsCRP |  |  |  | -2.03 | (-5.64, 1.58) | 0.27 |
| PC1 5C*Daily discrimination |  |  |  | 3.28 | (-1.83, 8.39) | 0.21 |
| PC1 5D*Age |  |  |  | -0.29 | (-0.73, 0.15) | 0.19 |
| PC1 5D*Female |  |  |  | 2.93 | (-5.83, 11.69) | 0.51 |
| PC1 5D*Polygenic risk score hsCRP |  |  |  | -0.60 | (-5.21, 4.00) | 0.80 |
| PC1 5D*Daily discrimination |  |  |  | 3.84 | (-1.65, 9.32) | 0.17 |
| PC1 5E*Age |  |  |  | -0.01 | (-0.53, 0.51) | 0.97 |
| PC1 5E*Female |  |  |  | 3.34 | (-7.41, 14.10) | 0.54 |
| PC1 5E*Polygenic risk score hsCRP |  |  |  | -2.75 | (-6.65, 1.16) | 0.17 |
| PC1 5E*Daily discrimination |  |  |  | 2.85 | (-2.85, 8.55) | 0.33 |
| Polygenic risk score hsCRP*Age |  |  |  | 0.00 | (-0.01, 0.01) | 0.57 |
| Polygenic risk score hsCRP*Female |  |  |  | 0.05 | (-0.12, 0.22) | 0.55 |
| Daily discrimination*Age |  |  |  | 0.00 | (-0.01, 0.01) | 0.49 |
| Daily discrimination*Female |  |  |  | 0.10 | (-0.08, 0.27) | 0.29 |
| Results were pooled across 10 multiple imputed datasets | | | | | | |
| hsCRP = High-sensitivity C-reactive protein; PC = principal component | | | | | | |

**Supplemental Table 3.** Multiple linear regression analysis of log-transformed high-sensitivity C-reactive protein levels among 729 Hispanic participants: Unstandardized coefficients for demographic factors, polygenic risk scores, principal components, daily discrimination experiences, and interaction terms. These results are from sensitivity analyses excluding body mass index.

| *Characteristic* | *b* | *95% CI* | *p value* | *b* | *95% CI* | *p value* |  |
| --- | --- | --- | --- | --- | --- | --- | --- |
| (Intercept) | 0.20 | (-0.39, 0.78) | 0.51 | 0.27 | (-1.09, 1.62) | 0.70 |  |
| Polygenic risk score hsCRP | 0.17 | (-0.02, 0.35) | 0.082 | 0.76 | (0.07, 1.45) | 0.030 |  |
| Daily discrimination | 0.11 | (0.02, 0.21) | 0.024 | 0.11 | (-0.69, 0.90) | 0.80 |  |
| PC1 5A | -5.99 | (-9.89, -2.09) | 0.003 | 3.16 | (-31.21, 37.53) | 0.86 |  |
| PC1 5B | 2.56 | (-1.54, 6.66) | 0.22 | 31.44 | (-1.08, 63.95) | 0.058 |  |
| PC1 5C | 1.36 | (-5.32, 8.04) | 0.69 | 18.95 | (-42.52, 80.43) | 0.55 |  |
| PC1 5D | -1.26 | (-4.96, 2.44) | 0.51 | -5.46 | (-37.05, 26.14) | 0.74 |  |
| PC1 5E | 1.85 | (-2.49, 6.19) | 0.40 | -0.80 | (-35.47, 33.87) | 0.96 |  |
| Age | 0.01 | (-0.01, 0.02) | 0.11 | 0.00 | (-0.02, 0.03) | 0.64 |  |
| Female sex (vs. Male) | 0.20 | (0.05, 0.35) | 0.009 | 0.40 | (0.05, 0.75) | 0.027 |  |
| Polygenic risk score hsCRP*Daily discrimination | -0.02 | (-0.13, 0.09) | 0.74 | 0.00 | (-0.13, 0.12) | 0.96 |  |
| PC1 5A*Age |  |  |  | -0.04 | (-0.54, 0.46) | 0.87 |  |
| PC1 5A*Female |  |  |  | -4.07 | (-12.40, 4.27) | 0.34 |  |
| PC1 5A*Polygenic risk score hsCRP |  |  |  | -1.07 | (-5.75, 3.60) | 0.65 |  |
| PC1 5A*Daily discrimination |  |  |  | -2.86 | (-8.54, 2.82) | 0.32 |  |
| PC1 5B*Age |  |  |  | -0.45 | (-0.89, -0.01) | 0.049 |  |
| PC1 5B*Female |  |  |  | 4.51 | (-3.94, 12.96) | 0.30 |  |
| PC1 5B*Polygenic risk score hsCRP |  |  |  | 1.78 | (-3.03, 6.59) | 0.47 |  |
| PC1 5B*Daily discrimination |  |  |  | -4.88 | (-10.99, 1.22) | 0.12 |  |
| PC1 5C*Age |  |  |  | -0.35 | (-1.20, 0.49) | 0.41 |  |
| PC1 5C*Female |  |  |  | -3.83 | (-17.95, 10.29) | 0.59 |  |
| PC1 5C*Polygenic risk score hsCRP |  |  |  | 4.02 | (-4.10, 12.15) | 0.33 |  |
| PC1 5C*Daily discrimination |  |  |  | 5.96 | (-4.79, 16.72) | 0.28 |  |
| PC1 5D*Age |  |  |  | 0.12 | (-0.34, 0.58) | 0.62 |  |
| PC1 5D*Female |  |  |  | -5.20 | (-12.69, 2.29) | 0.17 |  |
| PC1 5D*Polygenic risk score hsCRP |  |  |  | -3.36 | (-8.08, 1.35) | 0.16 |  |
| PC1 5D*Daily discrimination |  |  |  | 0.23 | (-4.63, 5.09) | 0.93 |  |
| PC1 5E*Age |  |  |  | 0.00 | (-0.44, 0.45) | 0.99 |  |
| PC1 5E*Female |  |  |  | -4.75 | (-13.80, 4.29) | 0.30 |  |
| PC1 5E*Polygenic risk score hsCRP |  |  |  | 3.95 | (0.20, 7.70) | 0.039 |  |
| PC1 5E*Daily discrimination |  |  |  | -0.25 | (-7.48, 6.98) | 0.95 |  |
| Polygenic risk score hsCRP*Age |  |  |  | -0.01 | (-0.02, 0.00) | 0.18 |  |
| Polygenic risk score hsCRP*Female |  |  |  | -0.32 | (-0.51, -0.14) | 0.001 |  |
| Daily discrimination*Age |  |  |  | 0.00 | (-0.01, 0.01) | 0.88 |  |
| Daily discrimination*Female |  |  |  | -0.12 | (-0.33, 0.08) | 0.23 |  |
| Results were pooled across 10 multiple imputed datasets | | | | | | | |
| hsCRP = High-sensitivity C-reactive protein; PC = principal component | | | | | | | |
